# Isolated Fallopian Tube Torsion in a Premenarchal Female Patient: A Case Report

**DOI:** 10.7759/cureus.93610

**Published:** 2025-09-30

**Authors:** Thuraya Al Kindi, Ibtisam H Al Shuaili, Saud S Shabibi

**Affiliations:** 1 Radiology, Oman Medical Speciality Board, Muscat, OMN; 2 Pediatric Radiology, Royal Hospital, Muscat, OMN

**Keywords:** acute pelvic pain, adnexal mass, gynecological emergency, isolated fallopian tube torsion, laparoscopy, ovarian torsion differential, paratubal cyst, pediatric gynecology, pelvic mri, preoperative diagnosis

## Abstract

Isolated fallopian tube torsion (IFTT) is a rare cause of acute pelvic pain, especially in pediatric patients. It is often misdiagnosed due to non-specific clinical and imaging findings. We report a 12-year-old girl who presented with acute onset of left lower abdominal pain and vomiting. Laboratory investigations, including tumor markers, were normal. Imaging studies (ultrasound and magnetic resonance imaging (MRI)) demonstrated a normal-appearing ovary with an adjacent heterogeneous adnexal mass. However, MRI findings raised suspicion for adnexal torsion. Emergency laparoscopy confirmed a 540-degree torsion of the left fallopian tube secondary to a paratubal cyst. The ovary was unaffected and appeared grossly normal. IFTT should be considered in adolescent females with pelvic pain and normal-appearing ovaries on imaging. Early surgical intervention is crucial for preserving fertility and preventing complications.

## Introduction

Fallopian tube torsion is most commonly recognized in association with ovarian torsion; however, Isolated fallopian tube torsion (IFTT), though rare, can occur independently and may present with similar clinical features [1]. IFTT is defined as complete twisting of the fallopian tube, occurring at least once, without involvement of the ovary. Congenital anomalies, such as hydrosalpinx, are thought to be predisposing factors [2]. It is recognized as a gynecological emergency, with an estimated incidence of one in every 1.5 million females. It is considered rare in children. Accurate preoperative diagnosis of IFTT is challenging because its symptoms are nonspecific and overlap with those of many other conditions, such as appendicitis and gastrointestinal causes [3-4]. While IFTT is seldom diagnosed preoperatively [5], recent advancements in ultrasound and other imaging techniques have significantly improved the ability to suspect it before surgery. If not promptly recognized and managed, IFTT can lead to infertility and ectopic pregnancy, further emphasizing the importance of timely diagnosis. In this case report, we sought to identify unique clinical or radiological signs that could suggest IFTT.

## Case presentation

The patient in our case, a 12-year-old girl, presented to the emergency department with a one-day history of left-sided abdominal pain, followed by vomiting. Moreover, she reported a history of constipation, for which she had been treated with laxatives over the past two days. Her last menstrual period occurred 10 days prior. She denied any urinary symptoms and had no fever. On examination, the child appeared unwell but was afebrile and vitally stable. Abdominal examination revealed a soft abdomen with tenderness localized to the left pelvic region. The remainder of the examination was unremarkable. The patient was admitted for workup under the differential diagnoses of left ovarian torsion, gastrointestinal causes, and, less likely, appendicitis, as the pain was primarily localized to the left iliac fossa.

Work up and imaging

**Figure 1:**
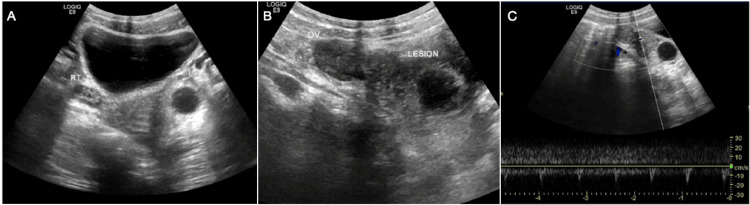
Ultrasound images of isolated fallopian tube torsion Image A shows a normal right ovary appearance and a cyst in the left adnexa. Image B shows the left adnexa, where the left ovary is noted to be pushed anteriorly with no obvious sonographic signs of torsion; posterior to it, there is a hypoechoic lesion containing an anechoic cyst. Image C shows vascularity within the lesion.

The patient had previously undergone an ultrasound at a private hospital, where a left adnexal cyst was reported. However, upon repeat ultrasound in our radiology department, the findings showed normal bilateral ovaries with no signs of torsion (Figure 1). The left ovary was displaced anteriorly by a heterogeneous structure or mass located posteriorly. This mass had an anechoic cystic component, with an adjacent echogenic area, which was suspected to contain fat. Small pelvic free fluid was also noted. 

Based on the ultrasound findings, there was no specific evidence of ovarian torsion, and the patient was admitted for further evaluation of a left adnexal mass. A teratoma was considered among the differential diagnoses, and tumor markers were sent for analysis. However, the tumor markers returned negative, and the remainder of her laboratory results were all within normal limits (Table 1).

**Table 1:** Laboratory reports of the patient HCG: human chorionic gonadotropin

Test	Result (normal range)
Complete blood count	
Hemoglobin	11.4 g/dL (11.5 – 15.5)
White blood cells	7.5 x 10^9^/L(4.5 – 14.5)
Platelet count	337 x 10^9^/L(150 – 450)
C reactive protein	<4 mg/L (<10 mg/L)
Rotavirus antigens	Negative
Feces microscopy	Negative for ova, cysts, and parasites
	Red cells not seen
	White cells not seen
Beta HCG	<2 iU/L (<5 iU/L for non-pregnant females)
Alpha fetoprotein	1.3 ug/L (0 – 20)
Lactate dehydrogenase	135 iU/L (120 – 246)

**Figure 2:**
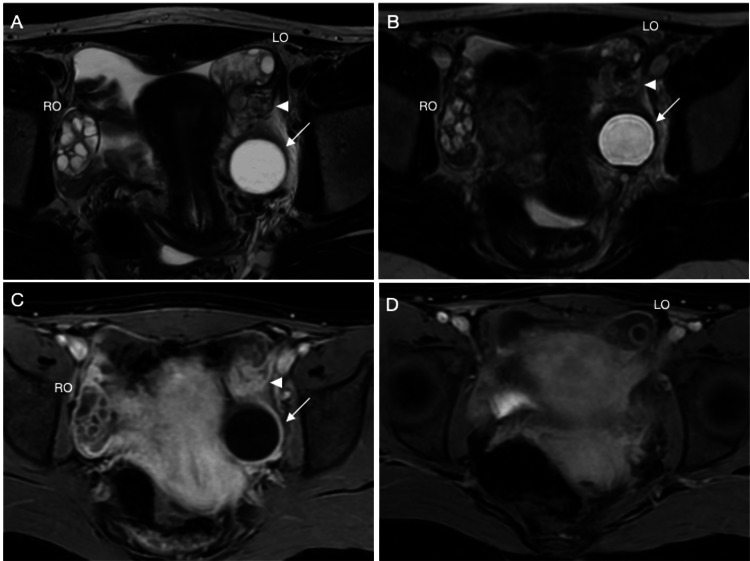
MRI T2 and post-contrast T1 images of isolated fallopian tube torsion T2 Images A and B in two consecutive levels show a normal right ovary with polycystic morphology, and the left ovary is pushed anteriorly with a parenchymal high T2 signal denoting edema. In the left adnexa posteriorly, there is a simple cyst (arrow); between the ovary and the cyst, there is a tortuous structure with an intermediate T2 signal (arrowhead), which turned out to be a twisted fallopian tube. Post-contrast T1 Images C and D in two consecutive levels show again the normally enhancing right ovary in Image C, and the left ovary shows follicular rim enhancement and peripheral enhancement of the left ovary as shown in Image D, which, along with edema, raised suspicion for a twisted left ovary. The twisted fallopian tube showed good enhancement, as shown in Image C.

Further imaging was performed with a pelvic magnetic resonance imaging (MRI) (Figure 2), which revealed a normal right ovary. The left ovary appeared slightly bulky, mildly edematous, and displaced anteriorly. A simple cyst was noted posterior to the left ovary, which was inseparable from it. Additionally, a thickened tubular structure with low T1 and T2 signals was observed in the left adnexa, adjacent to the edematous ovary and cystic structure. Based on these findings, the suspicion of left ovarian torsion with possible left fallopian tube torsion was raised, although the clear signs of ovarian torsion, such as gross enlargement of the ovary or cyst peripheralization, were absent. 

Management and final diagnosis

The patient was taken to the operating theater for emergency left ovarian detorsion with possible fallopian tube detorsion. Intraoperatively, the left fallopian tube was found to be twisted approximately 540 degrees. A cyst, measuring around 3 cm, was identified just proximal to the left fallopian tube fimbriae and was most likely the leading cause of the IFTT. The left ovary appeared completely normal, with no signs of torsion, though it was displaced anteriorly.

## Discussion

IFFT is a rare condition characterized by the twisting of the fallopian tube without involvement of the ovary. It is more commonly observed in reproductive-age women and considered rare in children.

Multiple etiologies have been identified as risk factors for IFTT. The most common cause is an excessively long mesosalpinx, which is the portion of the broad ligament that surrounds and supports the fallopian tubes, with fallopian tubes measuring more than 12 cm. Other risk factors include an incomplete distal mesosalpinx; hydrosalpinx, defined as serous fluid buildup in the ampulla from distal infundibular obstruction, with or without adhesions secondary to salpingitis; hematosalpinx, which refers to the accumulation of blood within the fallopian tube; and para-ovarian or tubal masses. In adolescents, the most common risk factor is congenital hydrosalpinx, often in conjunction with vigorous exercise [6].

There are no specific symptoms that point toward IFTT [6], which is the primary cause of delayed diagnosis and management. It typically presents with the same features as ovarian torsion, including moderate to severe pelvic pain, which may be diffuse or localized, and is often associated with nausea and vomiting [7].

Further testing and laboratory workup can help raise suspicion, but they remain non-specific, as leucocytosis and elevated C-reactive protein (CRP) can be observed in both adnexal torsion and appendicitis [1]. Tumor markers and human chorionic gonadotropin can provide additional value and help guide the differential diagnosis, especially in adult patients. In our case, the radiological findings, apart from the presence of a simple cyst, were not typical of teratoma, and the laboratory results supported this assessment.

Ultrasound is typically the first-line investigation. Certain features have been described as suggestive of fallopian tube torsion, including a normal ovary accompanied by a dilated, cystic-appearing tube with thickened and echogenic walls [1-2]. Doppler ultrasound has limited value, as the signal is often not detectable in the fallopian tube, and its presence does not exclude torsion. The whirlpool sign has been identified as specific for IFTT in the presence of normal ovaries; however, it is not consistently visualized [1]. The use of CT and MRI remains controversial, as both may delay surgical intervention and typically do not provide definitive findings for IFTT [1]. Nevertheless, in our case, the suspicion of IFTT was raised based on MRI findings.

Case reports were published by Mouad et al. (2024) and Toyoshima et al. (2015). Both described radiological findings of a dilated tubular structure consistent with hydrosalpinx complicated by IFTT. Another case by Toyoshima et al. reported a cystic mass with internal echogenic material. Our case was similar to the second case presented by Toyoshima, suggesting that mass lesions may predispose to torsion; however, the twisted fallopian tube in our case was not as markedly dilated as in previously reported cases. In the literature, IFTT has never been considered the primary differential diagnosis. Instead, ovarian torsion, appendicitis, and Meckel’s diverticulitis were suspected, with the latter ruled out by a negative Meckel’s diverticulum scintigraphy. In all reported cases, patients underwent exploratory laparoscopy after identification of a mass-like lesion with or without hydrosalpinx on ultrasound or MRI, and the diagnosis of IFTT was confirmed only intraoperatively [2,4].

Exploratory laparoscopy remains the gold standard for both the diagnosis and treatment of isolated fallopian tube torsion [2]. Conservative detorsion surgery is controversial, as it carries the risk of future infertility due to scarring, adhesions, and ectopic pregnancy. Additionally, IFTT is associated with a high recurrence rate [1]. However, one case series advocates for conservative management whenever feasible, given its significant impact on preserving future fertility [8].

## Conclusions

IFTT is a rare condition, especially in premenarchal girls, and its challenging diagnosis is a major factor contributing to delayed management. Therefore, in girls presenting with pelvic pain, IFTT should be included in the differential diagnosis, especially if there is an adnexal mass lesion with a dilated tubular structure (hydrosalpinx). When IFTT is strongly suspected, prompt emergency laparoscopy should be considered to salvage the fallopian tube and reduce the risk of future infertility. 
